# Equity in the provision of helicopter emergency medical services in the United Kingdom: a geospatial analysis using indices of multiple deprivation

**DOI:** 10.1186/s13049-024-01248-4

**Published:** 2024-08-20

**Authors:** Ryan D. McHenry, Caroline Leech, Ed B. G. Barnard, Alasdair R. Corfield

**Affiliations:** 1 ScotSTAR, Scottish Ambulance Service, Hangar B, 180 Abbotsinch Road, Paisley, PA3 2RY UK; 2 Emergency Department, University Hospitals Coventry & Warwickshire NHS Trust, Walsgrave, Coventry, CV2 2DX UK; 3 The Air Ambulance Service, Blue Skies House, Butlers Leap, Rugby, CV21 3RQ UK; 4 Academic Department of Military Emergency Medicine, Royal Centre for Defence Medicine (Research and Clinical Innovation), Birmingham, UK; 5 Department of Research, Audit, Innovation, and Development (RAID), East Anglian Air Ambulance, Norwich, UK

**Keywords:** Pre-hospital emergency medicine, Health inequalities, Geospatial modelling, Social deprivation, Delivery of health care

## Abstract

**Background:**

Helicopter Emergency Medical Services (HEMS) in the United Kingdom (UK) are provided in a mixed funding model, with the majority of services funded by charities alongside a small number of government-funded operations. More socially-deprived communities are known to have greater need for critical care, such as that provided by HEMS in the UK. Equity of access is an important pillar of medical care, describing how resource should be allocated on the basis of need; a concept that is particularly relevant to resource-intensive services such as HEMS. However, the Inverse Care Law describes the tendency of healthcare provision to vary inversely with population need, where healthcare resource does not meet the expected needs in areas of higher deprivation. It is not known to what extent the Inverse Care Law applies to HEMS in the UK.

**Methods:**

Modelled service areas were created with each small unit geography locus in the UK assigned to its closest HEMS operational base. The total population, median decile on index of multiple deprivation, and geographic area for each modelled service area was determined from the most recently available national statistics. Linear regression was used to determine the association between social deprivation, geographic area, and total population served for each modelled service area.

**Results:**

The provision of HEMS in the UK varied inversely to expected population need; with HEMS operations in more affluent areas serving smaller populations. The model estimated that population decreases by 18% (95% confidence interval 1–32%) for each more affluent point in median decile of index of multiple deprivation. There was no significant association between geographic area and total population served.

**Conclusion:**

The provision of HEMS in the UK is consistent with the Inverse Care Law. HEMS operations in more deprived areas serve larger populations, thus providing a healthcare resource inversely proportional with the expected needs of these communities. Funding structures may explain this variation as charities are more highly concentrated in more affluent areas.

**Supplementary Information:**

The online version contains supplementary material available at 10.1186/s13049-024-01248-4.

## Introduction

Helicopter Emergency Medical Services (HEMS) in the United Kingdom (UK) are provided in a mixed funding model, with the majority of services delivered by charities alongside a small number of government-funded operations. These services typically provide critical care at the site of serious illness or injury, with onward transport to hospital care [1].

The social determinants of health are well-established, with a clear association between social deprivation and higher incidence of disease, including critical illness and major trauma [2, 3], and poor health outcomes [4]. Most socially-deprived populations have higher requirements for critical care services, and worse outcomes in critical illness [3, 5]. HEMS have the potential to mitigate inequalities associated with social deprivation, with their focus on critical illness and geographic isolation, by delivering more rapid pre-hospital care in isolated areas.

The Inverse Care Law, first described in 1971, explains that the availability of good medical care tends to vary inversely with the need for it in the population served [6]. Provision of resource on the basis of need is at the core of healthcare equity, which also recognises social determinants as key drivers of healthcare requirements [4]. In a healthcare system that delivers equity, resources would be distributed not simply equally per capita, but according to expected need [7]. Such equity of access to HEMS might be seen if each resource served similar population numbers, or indeed, responded to the social determinants of health in its target population by concentrating services in areas of social deprivation or geographic isolation. The degree to which HEMS, a limited and expensive resource [8], are organised to best support their local communities is unknown.

The aim of this study was to quantify the current provision of UK HEMS relative to population, geographic area covered, and the degree of social deprivation experienced by populations served.

## Methods

The locations of HEMS operational bases in the UK were plotted using publicly available information [9]. Given the broad range of operational structures of HEMS in the UK, sites were included with no adjustment for level of care provided, such as physician-staffing, or diurnal variation in provision of care. Due to focus on HEMS, this analysis only considered aeromedical pre-hospital emergency care assets; dedicated interhospital transfer services and enhanced care services delivered by road were excluded.

Population and deprivation variables were derived from small area geographies available for each of the four UK nations, named Lower Layer Super Output Areas in England and Wales [10], Data Zones in Scotland [11], and Super Output Areas in Northern Ireland [12]. These local geographies were developed to allow dissemination of small area statistics, and while population numbers vary between nations, the geographies aim to contain similar population numbers within each nation. Each nation in the UK reports a separate area-based index of multiple deprivation, ranking these small area geographies by a number of metrics of social deprivation including employment, income, health, crime, education, and housing; which are often analysed as deciles [13]. This rank system allows the identification of deprived areas that might experience higher levels of unemployment, poorer housing quality, lower incomes, or more difficult access to services, or conversely of affluent areas where living standards might be higher. A lower number corresponds with a more deprived area.

**Fig. 1:**
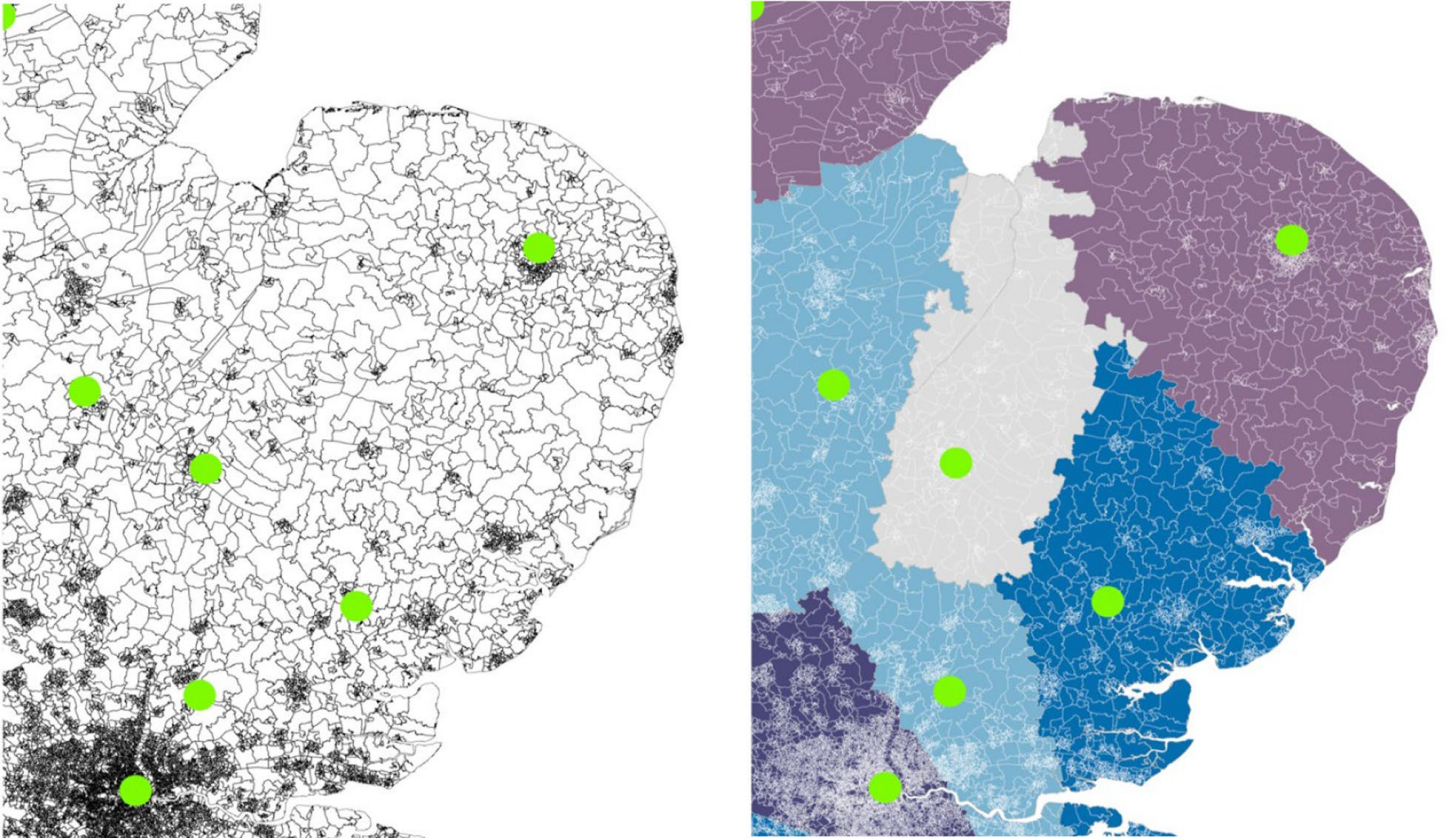
Plots demonstrating an example of the construction of modelled service areas. Left pane demonstrates the borders of small area geographies for the East of England with HEMS base locations (dots), and the right pane shows the distribution of each small area geography to its nearest HEMS resource (dots). Each area of uniform colour demonstrates the modelled service areas from which population statistics were extracted

The characteristics of the population served by each HEMS resource was determined with the construction of modelled service areas. These modelled service areas were created so that each small area geography in the UK was assigned to its nearest HEMS resource. Population statistics from small area geographies closest to each HEMS resource were then combined to give the total population, geographic area, and median decile on the index of multiple deprivation served by each HEMS resource. Population statistics were extracted from the most recently available iteration of national index of multiple deprivation statistics (Northern Ireland 2017, England and Wales 2019, and Scotland 2020) [10, 12, 14]. As HEMS organisations work closely with regional and national ground-based emergency medical services (EMS) [1], which are delivered independently in each of the four UK nations, the nearest local geography was determined on a national basis to best represent typical operations. The derivation of these modelled service areas, using the East of England as an example, is demonstrated in Fig. 1.

### Statistical analysis

Univariate and multivariate linear regression with log-transformation was used to assess the association between total population and both geographic area covered (in km^2^) and relative deprivation served by each operational base (defined by the median decile of index of multiple deprivation within each modelled service area), reported as coefficients with 95% confidence intervals (95% CI) and *p*-values, and visualised with a scatter plot.

Sensitivity analysis was undertaken with Spearman’s rank correlation coefficient for the same variables. A *p*-value < 0.05 was considered statistically significant. Choropleth mapping allowed identification of operational geographies by relative deprivation and geographic area. Analysis was undertaken using the osrm and sf packages in R (R Foundation for Statistical Computing, Vienna, Austria) [15, 16].

## Results

Thirty-six operational HEMS bases across the UK were identified, with two sites each hosting two operational daytime teams, giving 38 resources for analysis.

**Fig. 2:**
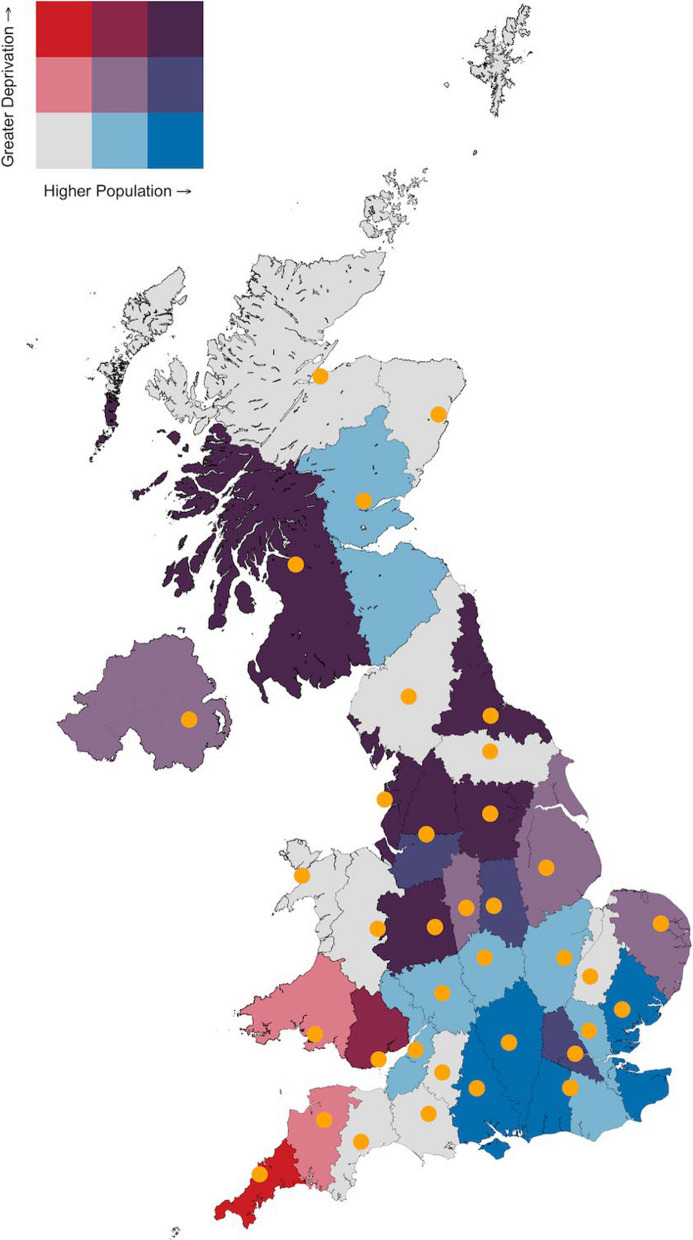
Bivariate choropleth map demonstrating the modelled service areas for each HEMS resource with population and socioeconomic characteristics. HEMS bases are indicated in orange. HEMS, helicopter emergency medical service

The median total population of the modelled service areas was 1,571,203 (range 304,465 to 8,636,383) and the median area served was 4555km^2^ (range 2,379 to 27,895km^2^). The full population characteristics for each modelled service area is seen in Supplementary Table 1. The area assigned to each operational HEMS resource, with population characteristics, is demonstrated in Fig. 2.

Univariate linear regression demonstrated a significant association between log-transformed total population and median decile of index of multiple deprivation, with smaller populations served in more affluent areas (R^2^ = 0.086, F(1, 36) = 4.48, *p* = 0.041).

**Fig. 3:**
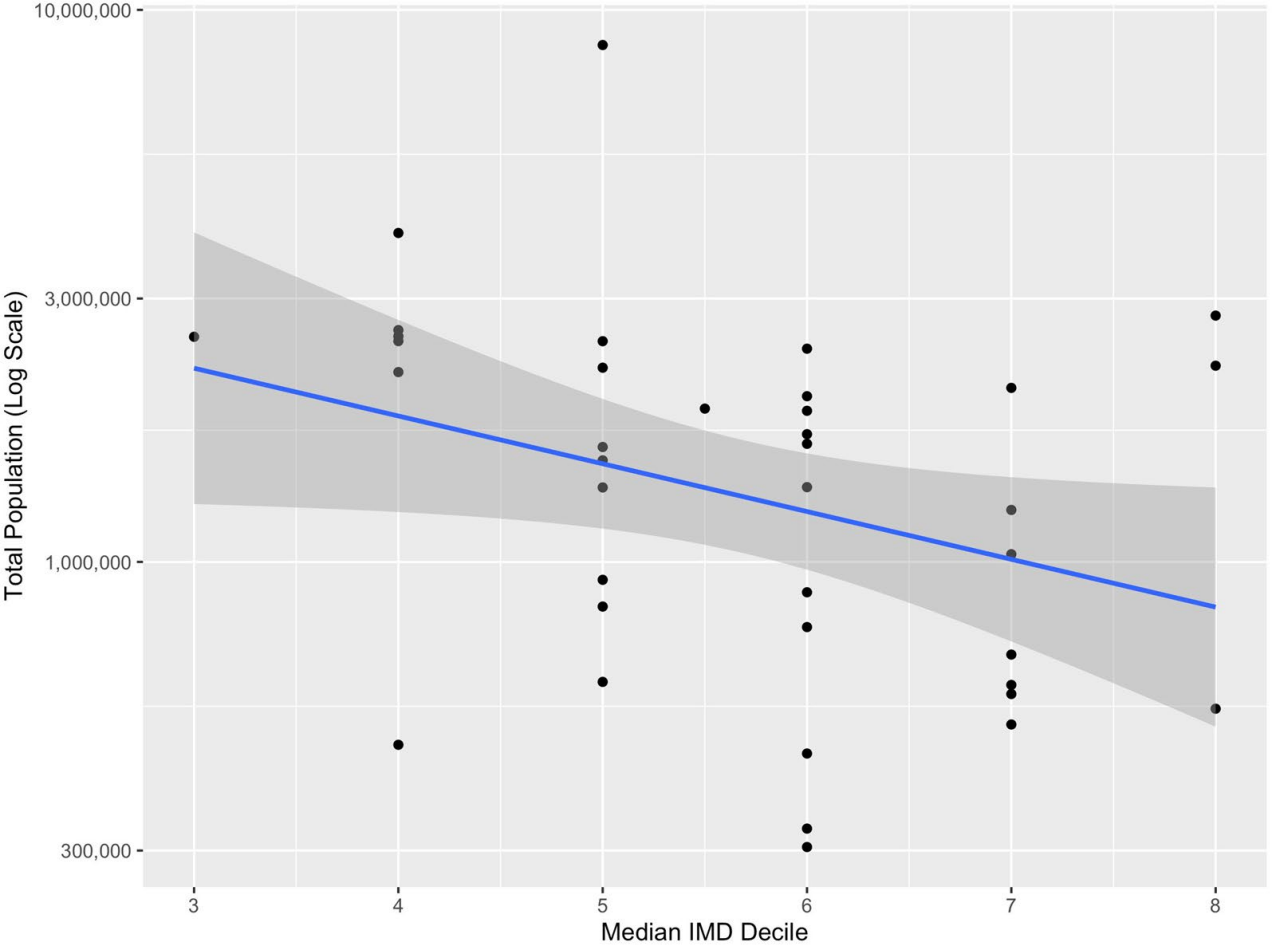
Scatter plot with linear regression line (blue) with 95% confidence interval (grey) for the univariate association between log-transformed total population and median index of multiple deprivation for each UK HEMS operational asset. HEMS, helicopter emergency medical service, IMD, index of multiple deprivation

The model estimated that population served by each HEMS resource decreases by 18% (95% CI 1–32%) for each point increase in median decile of index of multiple deprivation (Fig. 3).

On univariate analysis, no association was demonstrated between geographic area served and total population (R^2^ = − 0.008, F(1, 36) = 0.70, *p* = 0.41). The results of both univariate and multivariate analysis are demonstrated in Table 1.

**Table 1:** Results of univariate and multivariate linear regression for log-transformed total population of each HEMS modelled service area by median decile of index of multiple deprivation and log-transformed geographic area

	Univariate logistic regression estimate (95% Confidence Intervals)	*p* Value	Multivariate logistic regression estimate (95% Confidence Intervals)	*p* Value
Median Decile of Index of Multiple Deprivation	− 0.20 (− 0.39 to − 0.01)	0.041	− 0.21 (− 0.40 to − 0.02)	0.035
Area in km^2^ (log-transformed)	− 0.17 (− 0.58 to 0.24)	0.41	− 0.19 (− 0.58 to 0.20)	0.318

Spearman’s rank correlation coefficient demonstrated a negative correlation between population served and median index of multiple deprivation (Spearman’s Rho = − 0.38, *p* = 0.019), but no correlation between the size of area served and total population (Spearman’s Rho = − 0.081, *p* = 0.63)

## Discussion

The aim of this study was to quantify current provision of HEMS in the UK relative to population, geographic area covered, and the degree of social deprivation experienced by populations served. Delivery of HEMS in the UK is compatible with the Inverse Care Law, with provision varying inversely with expected population need just as documented in the original description of the law. Specifically, HEMS resources serving more deprived areas cover larger populations [6]. The total population served by HEMS resources in the UK was not associated with geographic area, an unexpected finding as it would seem logical that helicopter transport would have greater use where populations are more remote. Therefore, in a system delivering equity, more remote services might be expected to serve smaller populations. While these results are statistically and likely operationally significant, the models reflect only a small proportion of the variability observed.

The potential for more deprived areas to have greater need for HEMS or other pre-hospital critical care is consistent with evidence demonstrating the impact of socioeconomic status in pre-hospital care. For example, deprived areas have been shown to experience features associated with poorer outcomes, such reduced likelihood of bystander CPR, and features associated with greater requirement for advanced intervention, with more responses from pre-hospital critical care [17, 18]. The reduced availability of enhanced resource in more deprived communities has also been demonstrated for conventional ambulance transfer to advanced hospital care [19]. Together, these findings demonstrate a mis-match between the expected needs of communities and the resources available for their care.

These findings may be explained by funding structures. The majority of HEMS in the UK are charity funded, and there is evidence across the broader sector that such organisations are more prevalent in more affluent areas [20]. This hypothesis is supported by evidence of a public willingness to pay for helicopter medical services that outstrips their expected benefit in quality adjusted life years when compared to other medical interventions [21]. More affluent communities have greater resources to support such services [22]. These funding structures might also be seen as a continuation of the market forces that were thought to be the basis of Tudor Hart’s Inverse Care Law [6]. Historical factors related to the origins of HEMS in the UK might also have a role, while early services in Cornwall and Scotland served areas of relatively high deprivation, subsequent rapid expansion of services occurred in the relatively affluent South of England [23]. Another explanation may be the concentration of social deprivation in cities, less suited to HEMS operations compared to more rural areas. However, this explanation should be tempered by the presence of longstanding HEMS operations in major UK urban areas, our findings that area of coverage is not associated with populations served, and increasing suburbanisation of social deprivation [24, 25].

There are no other published data assessing the provision of HEMS across the UK by population and sociodemographic characteristics. The study methodology is strengthened by use of national level statistics, with accepted utility in determining allocation of healthcare resource [26], and geospatial modelling, with potential to define and quantify service areas and population characteristics.

However, this study does have limitations. Importantly, we assessed only the availability of helicopter transportation assets, not enhanced care teams, where availability might be by both road and air. No distinction was made between HEMS bases that provide solely pre-hospital critical care at the site of illness or injury, and those where availability for this role might be impacted by an additional remit for inter-hospital transfers. The decile of index of multiple deprivation was determined for each nation, giving a relative deprivation within each nation of the UK, but does not account for differences in the relative deprivation between nations. Assumptions were made that HEMS response to an area would typically be made by the closest resource, and may not reflect local service characteristics; including the occasional use of alternative base locations and standby points. While there is some evidence that areas experiencing higher deprivation have a greater requirement for pre-hospital critical care [18], the interventions that HEMS provides are complex and specialised, and it is not known to what extent requirements match area-level deprivation. We have used geographic and socioeconomic characteristics of the derived service areas as a marker of expected requirements for HEMS, and while these are known to influence the conditions to which HEMS responds [2, 3], other factors may also be important in determining the relative need of each population served.

Further work is required to assess the delivery of enhanced care services by ground assets, and how these services respond to the expected needs of more deprived populations. Policy should consider how to better distribute HEMS resource to better meet expected need, applying these findings and service planning methodology to mitigate health inequalities [27].

## Conclusion

The provision of HEMS in the UK is consistent with the Inverse Care Law. HEMS operations in more deprived areas serve larger populations, thus providing a healthcare resource inversely proportional with the expected needs of these communities. Funding structures likely explain this variation; charities are more highly concentrated in more affluent areas.

### Supplementary Information

Supplementary Material 1.

## Data Availability

Data are freely available. The analytical code is available from the author upon reasonable request.

## Abbreviations

HEMS: Helicopter emergency medical services
EMS: Emergency medical services
UK: United Kingdom
CI: Confidence intervals
IMD: Index of multiple deprivation
