# Supplementary material for: Equity in the provision of helicopter emergency medical services in the United Kingdom: a geospatial analysis using indices of multiple deprivation
Source: Scand J Trauma Resusc Emerg Med. 2024 Aug 20;32:73. doi: 10.1186/s13049-024-01248-4 (PMC11337590; doi:10.1186/s13049-024-01248-4)
Supplement: Supplementary file 1 — Supplementary Material 1. [file 13049_2024_1248_MOESM1_ESM.docx]

**Supplementary Table 1. Population characteristics, as median index of multiple deprivation and population, and area in km^2^ of each modelled service area.**

| **Modelled Service Area** | **Population** | **Median Index of Multiple Deprivation** | **Area in km^2^** |
| --- | --- | --- | --- |
| **1** | 1,365,970 | 6.0 | 3,434 |
| **2** | 2,557,607 | 3.0 | 3,232 |
| **3** | 2,247,956 | 5.0 | 2,897 |
| **4** | 2,207,424 | 4.0 | 2,980 |
| **5** | 679,445 | 7.0 | 3,417 |
| **6** | 1,703,746 | 6.0 | 3,774 |
| **7** | 449,710 | 6.0 | 10,689 |
| **8** | 606,279 | 5.0 | 4,923 |
| **9** | 2,433,677 | 6.0 | 6,278 |
| **10** | 2,511,003 | 5.0 | 3,499 |
| **11** | 881,071 | 6.0 | 4,036 |
| **12** | 762,010 | 6.0 | 3,848 |
| **13** | 1,636,583 | 6.0 | 5,433 |
| **14** | 466,412 | 4.0 | 2,881 |
| **15** | 1,996,159 | 6.0 | 2,379 |
| **16** | 2,629,854 | 4.0 | 6,319 |
| **17** | 928,041 | 5.0 | 5,881 |
| **18** | 3,943,382 | 4.0 | 5,194 |
| **19** | 542,096 | 8.0 | 2,419 |
| **20** | 2,793,892 | 8.0 | 6,262 |
| **21** | 2,563,178 | 4.0 | 5,696 |
| **22** | 598,680 | 7.0 | 5,099 |
| **23** | 2,266,934 | 8.0 | 3,053 |
| **24** | 1,242,094 | 7.0 | 3,090 |
| **25** | 8,636,383 | 5.0 | 2,415 |
| **26** | 1,032,646 | 7.0 | 4,186 |
| **27** | 1,364,262 | 5.0 | 2,824 |
| **28** | 2,066,719 | 7.0 | 5,316 |
| **29** | 1,614,991 | 5.0 | 9,024 |
| **30** | 304,465 | 6.0 | 3,750 |
| **31** | 1,527,415 | 5.0 | 3,654 |
| **32** | 830,101 | 5.0 | 6,922 |
| **33** | 507,605 | 7.0 | 6,460 |
| **34** | 576,390 | 7.0 | 7,584 |
| **35** | 2,512,023 | 4.0 | 25,428 |
| **36** | 328,773 | 6.0 | 27,894 |
| **37** | 1,878,217 | 6.0 | 17,042 |
| **38** | 1,895,506 | 5.5 | 14,149 |
